# Predictors of laminitis development in a cohort of nonlaminitic ponies

**DOI:** 10.1111/evj.13572

**Published:** 2022-04-01

**Authors:** Edward J. Knowles, Jonathan Elliott, Patricia A. Harris, Yu‐Mei Chang, Nicola J. Menzies‐Gow

**Affiliations:** ^1^ The Royal Veterinary College Hatfield UK; ^2^ Bell Equine Veterinary Clinic Mereworth UK; ^3^ WALTHAM Centre for Pet Nutrition Waltham on the Wolds UK

**Keywords:** adiponectin, horse, insulin, laminitis, PPID

## Abstract

**Background:**

Quantifying risk factors for laminitis development requires improvement.

**Objectives:**

To identify the most useful physical examination, metabolic and management factors to predict laminitis development in client‐owned, nonlaminitic ponies.

**Study design:**

Prospective cohort study.

**Methods:**

Physical examination, metabolic and management data were collected from a pony cohort 6 monthly for up to 4 years. Ponies were monitored for the development of laminitis. Metabolic data included basal plasma concentrations of ACTH ([ACTH]), adiponectin ([adiponectin]), triglycerides and glucose. Serum insulin concentrations ([insulin]) were measured in the unfasted basal state ([insulin]T0) and 60 minutes ([insulin]T60) after administration of corn syrup (0.3ml/kg). Separate multivariable Cox proportional‐hazards models were developed for physical, management/signalment and metabolic data and later combined into two final models. Low‐, medium‐ and high‐laminitis risk categories were defined based on basal or T60 [insulin].

**Results:**

Overall, 374 ponies (age 5‐32 years) and 891 pony‐years were included in the main analysis. Laminitis incidence (95% confidence interval (CI)) was 4.8 (3.5‐6.5) cases/100 pony‐years. Laminitis development was associated with numerous univariable factors. Significant (*P* < .05) factors retained in the final multivariable models included [insulin]T0, [insulin]T60, [adiponectin] and divergent hoof growth. [ACTH] was not independently associated with laminitis. Based on [Insulin]T0, low‐ (<21.6 µIU/ml), medium‐ (21.6‐45.2 µIU/ml) and high‐risk (>45.2 µIU/ml) categories encompassed 70, 20 and 10% of the population and had estimated 4‐year laminitis incidences (95%CI) of 6 (2‐9)%, 22 (10‐33)% and 69 (48‐82)% respectively. Based on [Insulin]T60 the low‐ (<53.4 µIU/ml), medium‐ (53.4‐153 µIU/ml) and high‐risk (≥153 µIU/ml) categories comprised 60, 30 and 10% of the population and had estimated 4‐year laminitis incidences (95%CI) of 3 (0‐6)%, 20 (10‐29)% and 73 (52‐84)% respectively.

**Main limitations:**

Results may not apply to different insulin assays, geographical regions, breeds or management types.

**Conclusions:**

[Insulin]T0 or [insulin]T60 best quantify the risk of future laminitis development in nonlaminitic ponies.

## Resumo

**Contexto:**

A quantificação dos fatores de risco para o desenvolvimento de laminite requere mais estudos.

**Objetivos:**

Identificar os fatores de exame físico, níveis metabólicos e de manejo mais úteis para predizer o desenvolvimento de laminite em pôneis não laminíticos de proprietários privados.

**Delineamento do estudo:**

Coorte prospectivo.

**Metodologia:**

Exame físico e dados metabólicos e de manejo foram coletados de um coorte de pôneis a cada seis meses por um máximo de quatro anos. Os pôneis foram monitorados para o desenvolvimento de laminite. Dados metabólicos incluíram: concentração basal de ACTH ([ACTH]), adiponectina ([adiponectina]), triglicérides e glicose. A concentração sérica de insulina ([insulina]) foi mensurada no estado basal sem jejum ([insulina]T0) e 60 minutos ([insulina]T60) após a administração de xarope de milho (0.3ml/kg). Modelos separados de riscos proporcionais de Cox multivariável foram desenvolvidos para dados de exame físico, manejo/identificação dos animais e metabólicos, que depois foram combinados em dois modelos finais. As categorias para o risco de desenvolvimento de laminite foram classificadas como baixa, média e alta, levando em consideração o nível basal ou T60 de insulina.

**Resultados:**

374 pôneis (idades entre 5 e 32 anos) e um total de 891 anos acumulados foram incluídos na análise. A incidência de laminite (95% de intervalo de confiança (IC)) foi 4.8 (3.5‐6.5) casos/100 anos. O desenvolvimento de laminite foi associado com inúmeros fatores invariáveis. Os fatores significantes (p<0.05) retidos no modelo de análise multivariável final foram: [insulina] T0, [insulina] T60, [adiponectina], e crescimento de casco divergente. [ACTH] não foi independentemente associado com laminite. Baseado nas categorias de [insulina] T0, as categorias de baixo (<21.6 µIU/ml), médio (21.6‐45.2 µIU/ml) e alto (>45.2 µIU/ml) risco foram compostas por 70, 20 e 10% da população e tiveram uma estimativa de 4 anos de incidência de laminite (95% CI) de 6 (2‐9)%, 22 (10‐33)% e 69 (48‐82%), respectivamente. Baseado na [insulina] T60, as categorias de risco baixa (<53.4 µIU/ml), média (53.4‐153 µIU/ml) e alta (≥153 µIU/ml) foram compostas por 60, 30 e 10% da população e tiveram uma estimativa de 4 anos de incidência (95% CI) de 3 (0‐6)%, 20 (10‐29)% e 73 (52‐84)%, respectivamente.

**Principais limitações:**

Esses resultados podem não ser aplicáveis com outras metodologias de mensuração de insulina, e outras regiões geográficas, raças ou tipos de manejo.

**Conclusões:**

[Insulina] T0 ou [insulina] T60 são os fatores que melhor quantificam o risco de futuro desenvolvimento de laminite em pôneis não laminíticos.

## 1 INTRODUCTION

Equine laminitis can be classified as endocrinopathic, inflammatory or mechanical in origin.^1^ Endocrinopathic laminitis is the predominant form in animals that present for lameness^1^, ^2^ and ponies are at higher risk of endocrinopathic laminitis than horses.^2^ Endocrinopathic laminitis has been associated with metabolic, morphometric and management risk factors.^3^, ^4^, ^5^ However, the relative importance of different risk factors and precise quantification of risk requires further study. A recent cohort study of ponies in Southern England reported a 1‐year baseline risk for the development of laminitis of 4%.^5^ The estimated 1‐year risk increased to 9.3% in ponies with increased basal serum insulin concentrations (>21.8 µIU/ml) and to 13.9% in those with low plasma adiponectin concentrations (<2.5 ng/ml).^5^ However, there was no benefit in combining these parameters into a clinical prediction model. Ponies in that cohort study were sampled once and management data were not collected.

The current study sought to improve the clinical prediction of the development of laminitis in a cohort of privately owned nonlaminitic ponies in the south‐east of England. It was hypothesised that the development of laminitis would be associated with management, metabolic and physical‐examination findings that could be combined into a clinical prediction model.

The specific aims were to:

1. Report the incidence of laminitis development.
2. Determine the most important physical, metabolic and management risk factors associated with laminitis development and combine these to improve clinical prediction.
3. Devise a risk categorisation that could be readily communicated to pony owners and clinicians.

## 2 MATERIALS AND METHODS

A prospective cohort study design was used. Each pony was visited every 6 months (spring and autumn) for up to eight visits (2015‐2019). Most spring visits occurred between 15 March and 15 April, and autumn visits between 15 September and 15 October. Ponies were followed‐up for the development of laminitis. For ease of analysis, visit dates were notionally assigned as 1 April and 1 October for spring and autumn respectively.

### 2.1 Animals

Pony‐owner recruitment and inclusion criteria have been reported previously.^6^ Briefly, eligible animals were ponies (≤149 cm with shoes), ≥5‐years of age with no owner reported history of likely or veterinary‐confirmed laminitis that were kept at a site with at least five eligible ponies. Ponies were excluded if they were pregnant, lactating, receiving pharmaceutical treatment for pituitary *pars intermedia* dysfunction (PPID) or if laminitis, significant other lameness or other disease was evident at the time of data collection. Ponies could enter the study at any data collection point and all eligible ponies (owner consent permitting) were included at each data collection time at each premises. If ponies were temporarily unavailable (eg away competing) at a data collection point they could be excluded from a period of analysis but re‐join the cohort subsequently. Although a survival analysis was planned, for practical reasons a sample size calculation was based on a binary/logistic analysis. The calculation assumed: an average follow‐up period of 2 years, a 2‐year laminitis incidence of 5%, a prevalence of 10%–27% and an odds ratio of 5‐10 for the main risk factor, β = 0.8 and, α = 0.05, and yielded an estimated requirement of 189‐447 ponies.

### 2.2 Data collection

At each visit each pony underwent a standardised veterinary examination. Three categories of data were collected: (i) blood analytes, (ii) physical/clinical measurements and (iii) an owner/keeper questionnaire.

#### 2.2.1 Blood analytes

Ponies were not fasted prior to sampling. They could be fed hay or kept at pasture, but owners were asked to withhold any additional complementary feed on that day. A basal blood sample was taken by jugular venepuncture into clot activator, EDTA, heparinised and oxalate fluoride vacutainer tubes (Becton Dickinson). Each pony was weighed with a portable weighbridge (Equestrian Weigh Platform, Equestrian Products) and a modified oral sugar test (OST) was performed. Ponies were syringe dosed 0.3 ml/kg Karo light corn syrup (ACH Food Companies Inc) orally to the nearest 50 kg. Rarely, ponies were not amenable to syringe dosing and were given the syrup in a small handful of low non‐structural carbohydrate feed. Blood samples were then taken by jugular venepuncture into clot activator vacutainer tubes 30 and 60 min (± 5 min) after corn syrup administration. Basal samples were analysed for serum insulin concentrations ([insulin]T0) and plasma concentrations of adiponectin ([adiponectin]), triglycerides ([triglycerides]), glucose ([glucose]) and adrenocorticotrophic hormone ([ACTH]). Samples taken at 30 and 60 min were analysed for insulin only ([insulin]T30 and [insulin]T60). Sample handling and assay details are included in Methods S1.

#### 2.2.2 Physical examination and morphometry

Ponies were examined (by EJK) to ensure they were healthy and the following physical parameters recorded: weight, height, body length, neck length, heart girth, belly girth, body condition score,^7^ cresty neck score,^8^ the presence or absence of hypertrichosis, bulging supraorbital fat pads, a pot belly or forelimb evidence of divergent hoof growth. Detailed definition of all variables considered for analysis is presented in Table S1.

#### 2.2.3 Management questionnaire

Owners/carers were asked to complete a questionnaire concerning the diet, management and health (including any PPID signs) for each pony at each visit (returned by post). Two questionnaires (see Data S1) were used, one at initial enrolment and another for each subsequent visit.

### 2.3 Outcome and follow‐up

The study was observational, and owners were asked to treat their ponies as usual. Owners were unaware of the results of blood analyses or the investigator's assessment of their ponies, although this could be requested for welfare reasons. At each visit owners/carers were asked about any suspected laminitis since the previous visit based on their opinion or that of their veterinary surgeon or farrier. Owners were contacted again by telephone or email 6 and 12 months after the final visit. If laminitis occurred, it was recorded as either owner‐suspected, farrier‐suspected or veterinary‐confirmed. Veterinary‐confirmed cases were diagnosed/confirmed as laminitis by a veterinary surgeon based on the clinical signs.

The study outcome was laminitis development (either veterinary confirmed or owner/farrier suspected), if this occurred the outcome date (to the nearest month) was recorded, data collected up to the date of laminitis were used for analysis and the pony then left the cohort immediately following laminitis development. If ponies moved premises or became ineligible (eg becoming pregnant), data were included up to the last known date that the pony remained eligible for inclusion. Follow‐up for all cases ceased in spring 2020.

### 2.4 Data processing and analysis

#### 2.4.1 Data processing

Data were checked and processed using database software (Filemaker Pro 12, Claris International Inc and Microsoft Excel). Missing values were not usually imputed; however, for blood analytes other than [ACTH], the previous sampling data point was carried forward if a value was missing. Missing blood analyte data occurred rarely and was usually due to pony behaviour. If questionnaires were not returned or completed properly owners were asked by telephone or email for signalment data but were not asked to remember other management details. Missing data were imputed for four questionnaire variables only under specific circumstances (Table S1).

Due to the normal seasonal variation in [ACTH]^9^ and presence of autumnal cross‐reactants,^6^ spring and autumn [ACTH] absolute values were not included in the main analysis. An additional binary variable “ACTH positive” was created to categorise ponies into those with or without [ACTH] that exceeded the middle of the equivocal range proposed by the Equine Endocrinology Group Guidelines for PPID diagnosis^10^ in autumn and spring (75 and 40 pg/ml respectively).

Prior to analysis, data were processed into biologically plausible variables that were considered potential contributors to laminitis. In some cases, composite variables were created to reduce the total number of variables evaluated and so the chance of type 1 errors. Where appropriate, the number of levels of categorical variables was minimised. Ordinal variables with at least five categories such as body condition score were analysed as continuous variables after assessing the data distribution and linearity.^11^ For illustrative purposes [ACTH] and [insulin]T60 were rescaled (divided by 10) for parts of the analysis ([ACTH]_10_ and [insulin]T60_10_ to illustrate the increase in laminitis hazard for every 10pg/ml or 10µIU/ml rather than every 1pg/ml or µIU/ml. A list of variables considered for analysis and their definitions is included in Table S1.

#### 2.4.2 Data analysis

Data were analysed using statistical software (R 4.0.3, R Foundation for Statistical Computing). The main analysis assessed a 4‐year period at risk (1/10/2015‐30/9/19). The incidence of laminitis was calculated by dividing the number of episodes of laminitis by the total time at risk. Rates were compared for the summer (spring to autumn) and winter (autumn to spring) using a rate ratio test and assuming a Poisson distribution.

To analyse the risk factors for laminitis development, total time at risk was divided into nonoverlapping 6‐month period accounting for changes in the predictor variables during the period at risk (time‐dependent covariates).^12^ Eight 6‐month periods were used, each beginning with a data collection visit. A limited number of variables were selected from the possible list of predictors to create four prespecified initial multivariable time‐dependent Cox proportional‐hazards models to investigate associations between different predictor types and laminitis development hazard. The variable types used to create the four initial models were: (i) morphometric and clinical examination variables, (ii) owner questionnaire and signalment variables, (iiia) basal blood analytes and (iiib) blood analytes including [insulin]T60_10_ rather than [insulin]T0.

The variables for inclusion in each initial model were selected based on biological plausibility, practicality of data collection (to facilitate model application) and with the aim of reducing the numbers of correlated predictors in each model. To ensure a minimum of five events per variable, initially each model was restricted to six variables.^13^ Blood analyte models were created with and without the OST to indicate any benefit of the OST compared with basal samples. In order to reduce overfitting associated with excessive iterative modelling,^14^ all selected variables were entered into each initial model and no variables were eliminated.

After creating the initial models, two combined models were made, using predictor variables from each initial model. One combined model included only basal blood values, the other included [insulin]T60_10_ rather than [insulin]T0. Variables were eligible for inclusion if they were associated with a *P* value of < .1 in the initial (prespecified) multivariable models (for blood analytes a *P* < .1 was required in both initial models). Variables were removed by backward elimination until only those associated with *P* < .157 (based on the Akaike information criteria)^15^ were included in the final model.^16^ Model fit was assessed by concordance and the R^2^ of explained variation,^17^ a value analogous to the *R* ^2^ commonly reported for linear regression models. Concordance indicates the proportion of patient/pony pairs (within each 6‐month time period) in which the model predictions and observed outcomes agree and is equivalent to the area under the ROC curve for binary outcomes.^18^ Comparison of goodness‐of‐fit between multivariable models and selected single predictor models was evaluated descriptively using concordance measures, formal concordance comparisons were not performed due to small differences in the data available for each model. For all multivariable Cox proportional‐hazards models, the proportional‐hazards assumption was assessed by analysis of the Schoenfeld residuals. Due to limited laminitis cases, interactions between the main effects were not tested, nor was the effect of clustering of the covariates. For illustration, univariable, time‐dependent Cox proportional‐hazards models were also created for all plausible variables (without correction of *P* values).

The main predictor variables were divided into categories and survival curves for time‐dependent covariates were plotted.^19^ Nonparametric estimates of the cumulative laminitis incidence were calculated^19^ together with time‐dependent Cox proportional‐hazard models with each variable analysed as a factor. A subjective approach was taken to determine the risk categories based on the stratification of risk across the deciles of each variable for illustrative purposes.

A different approach was required for [ACTH] analysis as a continuous variable. Separate time‐dependent Cox‐proportional‐hazards models were created for spring and autumn samples. This analysis required an additional 6 months of follow‐up such that 4‐, 12‐month periods could be analysed for the autumn and spring separately (1/10/2015‐30/9/2019 and 1/4/2016‐31/3/2020). Univariable associations were initially assessed between [ACTH] and laminitis hazard. To determine whether any association between [ACTH] and laminitis was a proxy for insulin dysregulation, [insulin]T0 or [insulin]T60 were added to the spring and autumn [ACTH] models.

## 3 RESULTS

The study included 24 premises and 374 ponies of which 216 were geldings, 147 mares and two stallions (sex data were missing for nine ponies). The mean age (±standard deviation [SD]) during the time at risk was 14 (±6.3) years. Breed data were available for 291 ponies (105 Welsh or Welsh crosses, 48 Shetlands or Shetland crosses, 58 Cobs or Cob crosses and 80 “other breeds”). In total, 252 ponies were used for general riding, 75 as pets/retired, 10 for competition, five for breeding, 11 for driving and 31 for other or unspecified purposes.

### 3.1 Laminitis incidence

The main follow‐up period (1/10/2015‐30/9/2019) included 891 pony‐years at risk during which 43 cases of laminitis were reported, of which 32 were veterinary‐confirmed. Of the remaining 11 cases, the laminitis was diagnosed by a farrier in eight and by the owner/carer (all experienced riding school owners/managers) in three. A total of 128 ponies left the cohort for reasons other than laminitis, of which 33 died or were subjected to euthanasia and 70 were moved or sold. Other reasons for leaving the study included exclusion for behavioural reasons (n = 8), becoming pregnant (n = 3) or starting pergolide treatment (n = 5). The number of ponies included at each time period, the number of cases that developed laminitis and the incidence of laminitis are shown in Table 1. The incidence of laminitis was 3.23 (1.64‐7.14 (95% C.I)) times lower in winter than summer (*P* < .001).

**TABLE 1:** Number of ponies assessed at each data collection point, pony‐years of follow‐up, laminitis cases, ponies lost from the cohort and laminitis incidence during the main study period 1/10/2015‐30/9/2019)

Year	Season	Ponies assessed (of which n were newly recruited)	Laminitis cases	Pony‐ years at risk	Nonlaminitic ponies that left the cohort			
					Moved premises	Died/ euthanasia	Started pergolide treatment	Other^a^
2015	Autumn	207 (207)	2	101	11	2	1	1
2016	Winter							
	Spring	239 (51)	7	115	12	3	0	1
	Summer							
	Autumn	242 (28)	2	117	10	4	0	2
2017	Winter							
	Spring	238 (19)	5	115	8	3	2	5
	Summer							
	Autumn	250 (32)	5	119	3	5	1	2
2018	Winter							
	Spring	237 (11)	11	112	11	6	1	5
	Summer							
	Autumn	223 (11)	1	105	6	4	0	2
2019	Winter							
	Spring	232 (15)	10	107	9	6	0	2
	Summer							
Totals		1868	43	891	70	33	5	20
Incidence			4.8 (3.5‐6.5) Cases/100 pony years (95% CI)					
Summer (spring‐ autumn)			33	449				
Incidence			7.3 (5.1‐10.3) Cases/100 pony years (95% CI)					
Winter (autumn‐spring)			10	442				
Incidence			2.2 (1.1‐4.1) Cases/100 pony years (95% CI)					

^a^ Other reasons for leaving the cohort included (n): pony behaviour (8), pregnancy (3), owner or yard withdrew (6), unwell (then subjected to euthanasia) (2), unrecorded (1).

### 3.2 Risk factors for laminitis (main analysis)

The complete rate, data distribution (and central tendency for continuous variables) are shown in Table S2 for variables collected as potential predictors of laminitis. The initial time‐dependent Cox proportional‐hazards models are shown in Table 2. For the model using the OST, [Insulin]T60 was selected over [insulin]T30 as the mean response at T60 was larger. The model using blood analytes with [insulin]T60 had the highest concordance (and highest R^2^ of explained variation). All models contained factors with statistically significant hazard ratios. Significant contributors to laminitis risk included a higher body condition score, evidence of divergent hoof growth, the presence of hypertrichosis on veterinary examination, increasing age, higher [insulin]T0 and [insulin]T60, lower [adiponectin] and lower exercise levels and/or intensity. The combined time‐dependent Cox‐proportional‐hazards models are shown in Table 3. All variables included in the combined models were retained other than the presence of veterinary‐assessed hypertrichosis and age. There were slight differences in the fit of the two combined models, but the concordance values suggest no significant difference in predictive power. Univariable analysis of the statistically significant predictor variables from the combined models is shown in Table 4 and suggests that little additional predictive power (as indicated by model concordance) was achieved by the combined multivariable models compared with assessing only a single blood analyte (in particular [insulin]T60). For illustration, univariable, time‐dependent Cox proportional hazard models for all variables considered for inclusion in the analysis are shown in Table S3.

**TABLE 2:** Initial time‐dependent Cox proportional‐hazard models of factors associated with the laminitis development: Panel A: 1: morphometry and clinical examination variables; 2: questionnaire and signalment variables. Panel B: 3a basal blood analytes. 3b basal bloods analytes with [insulin]T60 substituted for [insulin]T0. NB [insulin]T60 was divided by 10

Panel A				
Variable	Hazard ratio	Lower .95	Upper .95	*P*=
Model 1: morphometry and clinical examination				
Body condition score^a^	1.47	0.96	2.26	.08
Cresty neck score (CNS)	1.29	0.88	1.87	.2
Height	0.99	0.97	1.01	.3
Hoof divergence score^b^	1.83	1.35	2,471	<.001
Hypertrichosis^c^	5.35	1.7	16.87	.004
Concordance	= 0.759 (se = 0.033)			
Wald test	= 32.36 on 5 *df*, *P* <.001			
*R* ^2^ (mev)	= .45			
n = 1572, number of events = 37 (296 observations missing)				
Model 2: questionnaire and signalment				
Shetland or Shetland X	0.57	0.18	1.81	.3
Other breed/Unknown	0.81	0.34	1.91	.6
Cob/Cob X	1.98	0.73	5.34	.2
Age	1.06	1.01	1.12	.03
Sex = female	1.40	0.70	2.80	.3
Exercise composite score^d^	0.81	0.72	0.91	<.001
Footsore after trimming^e^	0.00	0.00	Inf	>.9
Turnout composite score^f^	0.99	0.86	1.14	.9
(breed reference category = Welsh/Welsh X)				
(sex reference category = male)				
Concordance	=0.745 (se =0.041)			
Wald test	= 21.16 on 8 *df*, *P* =.007			
*R* ^2^ (mev)	=.37			
n = 1394, number of events = 33 (474 observations missing)				

Panel B				
Variable	Hazard ratio	Lower .95	Upper .95	*P*=
Model 3a ‐ basal blood				
[Adiponectin]	0.91	0.87	0.96	<.001
[Glucose]	0.89	0.55	1.45	.7
[Triglycerides]	1.92	0.85	4.34	.1
ACTH positive	0.56	0.2	1.52	.3
[Insulin]T0	1.01	1.00	1.01	<.001
Concordance	= 0.8 (se =0.04)			
Wald test	= 58.88 on 5 *df*, *P* < .001			
*R* ^2^ (mev)	=.61			
n = 1814, number of events = 42 (54 observations missing)				
Model 3b ‐ basal blood with oral sugar test insulin				
[Adiponectin]	0.94	0.90	0.98	<.001
[Glucose]	0.97	0.66	1.43	.9
[Triglycerides]	0.96	0.31	2.96	>.9
ACTH positive	0.42	0.15	1.15	.09
[Insulin]T60_10_ ^g^	1.07	1.05	1.09	<.001
Concordance	= 0.837 (se = 0.038 )			
Wald test	= 98.11 on 5 *df*, *P *≤ 2 × 10^‐16^			
*R* ^2^ (mev)	= .79			
n = 1801, number of events = 42 (67 observations missing)				

Note

Further detail of the variables is provided in Table S1.

Abbreviations: NB n, the number of 6‐month period analysed; *R* ^2^(mev) = *R* ^2^ of explained variation.

^a^ Overall body condition score (arithmetic mean of constituent scores).

^b^ Subjective veterinary assessment of divergent growth visible on the lateral aspects of the front feet.

^c^ Subjective veterinary assessment of hypertrichosis (present/absent).

^d^ A composite score to combine the owner‐reported intensity and frequency of exercise.

^e^ Owner reported history of the pony being footsore after foot trimming/shoeing during previous 3 months (yes/no).

^f^ A composite score to combine the owner‐reported duration of turnout and perceived extent of grass cover.

^g^ Serum insulin concentration 60 min after oral administration of 0.3 ml/kg Karo corn syrup, concentrations were divided by 10 to rescale and indicate the hazard ratio associated with each 10 µIU/ml increase.

**TABLE 3:** Time‐dependent Cox proportional‐hazard models of factors associated with laminitis development. Models were created following backward variable elimination after combining the initial models

Variable	Hazard ratio	Lower .95	Upper .95	*P*=
Combined model 1 with insulinT0 (basal)				
Exercise composite score^a^	0.90	0.80	1.02	.09
[Adiponectin]	0.94	0.89	0.98	.008
[Insulin]T0	1.005	1.00	1.01	.02
Body condition score^b^	1.31	0.97	1.77	.08
Hoof divergence score^c^	1.52	1.14	2.02	.004
Concordance	= 0.816 (se =0.037)			
Wald test	= 61.15 on 5 *df*, *P* < .001			
*R* ^2^ (mev)	= .67			
n = 1508, number of events = 37 (360 observations missing)				
Combined model 2 with insulinT60				
Exercise composite score^a^	0.92	0.81	1.03	.1
[Adiponectin]	0.96	0.92	1.01	.1.
[Insulin] (T60)_10_ ^d^	1.05	1.03	1.07	<.001
Body condition score^b^	1.29	0.94	1.78	.1
Hoof divergence score^c^	1.35	1.01	1.81	.04
Concordance	= 0.84 (se =0.036 )			
Wald test	= 87.66 on 5 *df*, *P* < .001			
*R* ^2^ (mev)	= .77			
n = 1502, number of events = 36 (366 observations missing)				

Note

NB *n*, the number of 6‐month periods analysed; *R* ^2^(mev), *R* ^2^ of explained variation.

Further detail of the variables is provided in Table S1.

^a^ A composite score to combine the owner‐reported intensity and frequency of exercise.

^b^ Overall body condition score (arithmetic mean of constituent scores).

^c^ Subjective veterinary assessment of divergent growth visible on the lateral aspects of the front feet.

^d^ Serum insulin concentration 60 min after oral administration of 0.3 ml/kg Karo corn syrup, concentrations were divided by 10 to rescale and indicate the hazard ratio associated with each 10 µIU/ml increase.

**TABLE 4:** Univariable time‐dependent Cox proportional‐hazards models to indicate associations between the main predictor variables identified in the composite models and the laminitis hazard

Explanatory Variable	Hazard ratio (95% CI)	P value (Wald)	Concordance (95% CI)
[Insulin]T60_10_	1.08 (1.06‐1.09)	<0.001	0.84 (0.77‐0.91)
[Insulin]T0	1.01 (1.01‐1.01)	<0.001	0.79 (0.72‐0.86)
[Adiponectin]	0.88 (0.84‐0.93)	<0.001	0.77 (0.69‐0.85)
Hoof divergence score	1.9 (1.4‐2.5)	<0.001	0.68 (0.61‐0.75)

Note

CI = confidence interval (estimated as ±1.96 × standard error for concordance).

**FIGURE 1:**
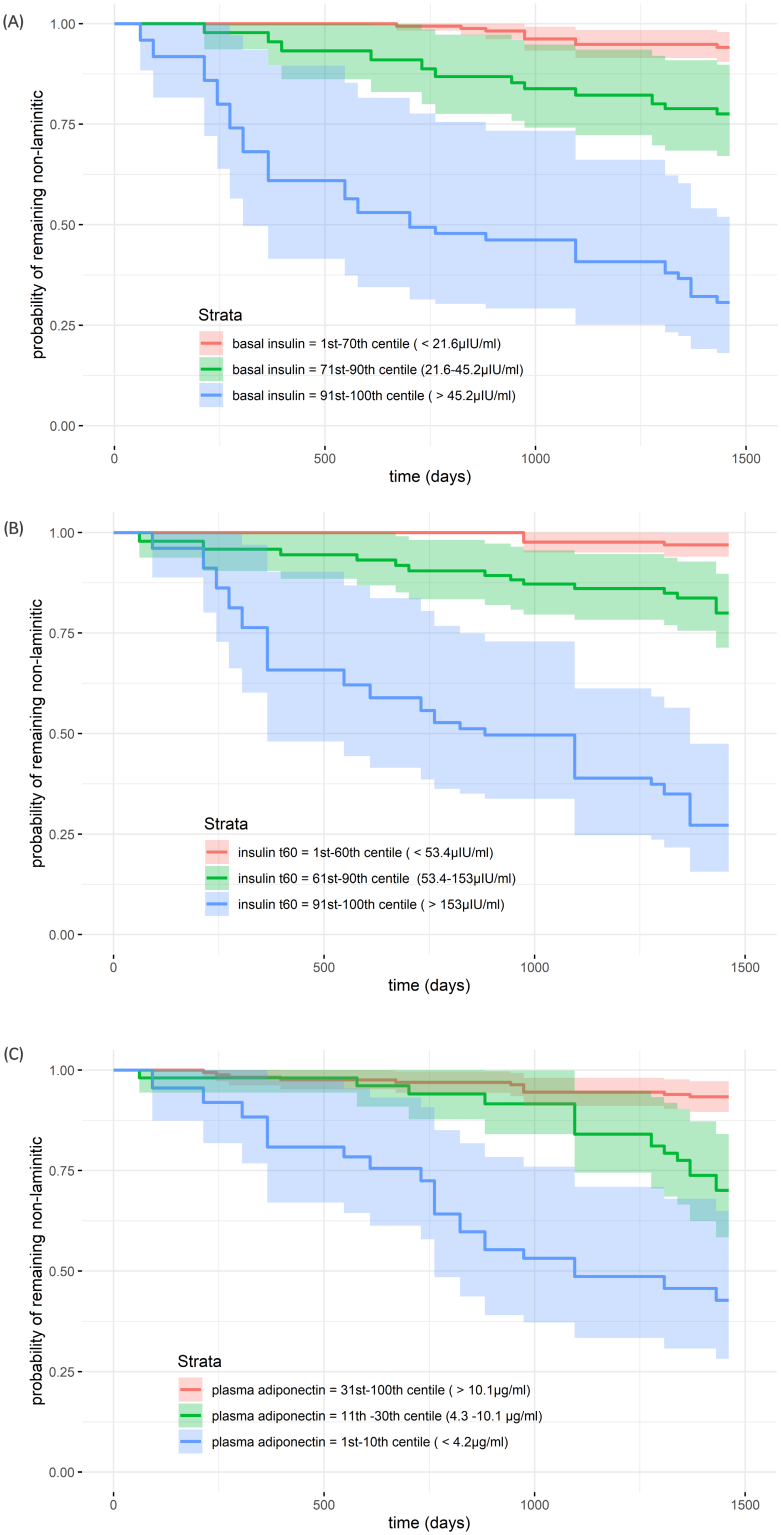
Survival curves^19^ for time‐dependent covariates to illustrate the estimated probability of remaining nonlaminitic for low‐, medium‐ and high‐risk categories [insulin]T0 (basal insulin} (panel A), [insulin]T60 (panel B) and [adiponectin] (panel C). 95% confidence intervals are shaded

Plots to illustrate laminitis risk in different categories (low, medium and high risk) of the main blood analyte predictor variables are shown in Figure 1. The estimated hazard ratios associated with each risk category, and 4‐year cumulative incidence are shown in Table 5.

**TABLE 5:** Estimated cumulative laminitis incidence, hazard ratios and centiles of the population for low‐, medium‐ and high‐risk categories for [insulin]T0, [insulin]T60 and [adiponectin]

Analyte	Risk category (concentration)	Centiles	Hazard ratio (Ref = reference category)	Cumulative incidence of laminitis (%) (95% CI)			
				1 y	2 y	3 y	4 y
[insulin]T0 µIU/ml	Low <21.6	0‐70th	Ref	NA	1 (0‐2)	5 (2‐8)	6 (2‐9)
	Medium 21.6‐45.2	71st‐90th	4.2 (1.8‐10.1)	4 (0‐10)	11 (1‐20)	18 (6‐ 28)	22 (10‐33)
	High >45.2	91st‐100th	18.6 (8.5‐40.8)	39 (10‐58)	51 (22‐69)	59 (34‐75)	69 (48‐82)
[insulin]T60 µIU/ml	Low <53.4	0‐60th	Ref	NA	NA	2 (0‐5)	3 (0‐6)
	Medium 53.4‐153	61st‐90th	7.6 (2.5‐23)	4 (0‐10)	9 (2‐17)	14 (5‐22)	20 (10‐29)
	High >153	91st‐100th	44.3 (15.2‐128.7)	34 (10‐52)	44 (9‐41)	61 (39‐75)	73 (52‐84)
[adiponectin] µg/ml	Low >10.1	31st‐100th	Ref	2 (0‐4)	3 (0‐6)	5 (2‐9)	7 (3‐10)
	Medium 4.3‐10.1	11th‐30th	5 (2.3‐11)	2 (0‐6)	6 (0‐12)	16 (5‐26)	30 (16‐42)
	High <4.2	0‐10th	12.1 (5.6‐25.9)	19 (3‐33)	27 (9‐42)	51 (29‐67)	57 (35‐72)

Note

NA = no events, survival not calculated. All groups were differed significantly from the reference category (*P* ≤ .001) using time‐dependent Cox proportional‐hazards models.

### 3.3 Risk factors for laminitis (ACTH analysis)

Univariable analysis of the 6‐month periods during the main follow‐up period revealed no significant association between either absolute [ACTH] or dichotomised “ACTH positive” variable and laminitis. During the additional 6 months of follow‐up (1/10/19‐31/3/20) required for separate analysis of spring and autumn ACTH, one additional case of laminitis was suspected (by an experienced owner). There was no association between [ACTH] and laminitis for samples taken in the spring (*P* = .1). In the autumn, [ACTH]_10_ (divided by 10 to rescale) was associated with a small increased risk of laminitis (estimated hazard ratio (95% CI) of 1.02 (1.00‐1.03) (*P* = .009)). However, when [insulin]T0 was added to the models, an independent association between autumn [ACTH] and laminitis risk was unlikely and not statistically significant (*P* = .1 and *P *≥ .9 for autumn and spring respectively). Similar results occurred if [insulin]T60 was added to the models (*P* = .9 and *P* = .4 for autumn and spring respectively).

## 4 DISCUSSION

The incidence of laminitis in the current study of 4.8 cases per 100 pony years is consistent with a previous study^5^ and indicates the significant burden of laminitis in such pony populations. Inclusion of owner/farrier‐suspected laminitis cases was supported by a recent study demonstrating the specificity (but not sensitivity) of owners to recognise laminitis.^20^ Furthermore, the owners reporting laminitis were all experienced horse keepers. However, the rate of laminitis reported in the current study is likely to be an underestimate of the true laminitis incidence as a proportion of cases may have gone unnoticed and only first occurrences of laminitis were included. Thus, had recurrent and mild/subclinical cases been included, the total laminitis burden would likely be higher than the current estimate.

The marked seasonal difference in laminitis incidence in which summer rates were approximately three times higher than winter rates is consistent with previously reported seasonal patterns in some studies,^21^, ^22^, ^23^ but contrasted with some other reports.^24^, ^25^ Season could not be included in the Cox proportional‐hazards models as ponies could not experience different seasons at the same time. However, the differences found indicate season is an important factor in disease prediction. The specific seasonal changes that result in a higher laminitis incidence were not determined, although changes in pasture nutritional content are likely contributors.^26^ In the current study, the “turnout composite score” variable to reflect time and owner reported subjective quality of turnout was not a useful predictor of laminitis. However, the accuracy of turnout data was limited by the time between data collection and laminitis development and the lack of objective quantification of pasture quality and intake. Further, this analysis may have been biased if owners altered turnout for ponies they perceived to be at higher risk of laminitis. Pasture/dietary changes alone do also not fully explain the seasonal effects on laminitis incidence as laminitis prone ponies show different seasonal metabolic changes when compared with nonlaminitic herd‐mates grazing the same pasture.^27^

Consistent with the authors’ hypothesis, a wide range of variables were associated with the development of clinically apparent laminitis and each initial multivariable model had some predictive power. Many of these predictor variables are likely to be collinear or confounding. In the combined final models, [insulin]T0, [insulin]T60, [adiponectin] and hoof divergence were all significantly associated with laminitis; however, none of the models had a higher concordance than [insulin]T60 alone and this was not materially superior to [insulin]T0. Therefore, despite the apparent multifactorial nature of laminitis, in the current study [insulin] was the primary laminitis risk indicator, with relatively little benefit gained by including the other factors assessed. However, the difficulty in recording and quantifying dietary factors that could have played a role at the time that the laminitis occurred may be a significant limitation.

Division of continuous variables [insulin]T0, [insulin]T60 and [adiponectin] into categories provided a practical illustration of the data, and the risk distribution was striking. Rather than increasing linearly over the population, the risk of laminitis was concentrated in a small proportion of ponies, perhaps consistent with a threshold effect. It must be acknowledged that the three‐group categorisation was based on subjective risk stratification rather than an objective analysis, such as dichotomisation based on a ROC curve. However, this categorisation was considered more biologically plausible and clinically intuitive. The categorical analysis also facilitates comparisons with previous studies. For example, the hazard ratios between low risk‐ and either the medium‐ or high‐risk groups for [insulin]T0 are within the 95% confidence intervals of the odds ratio reported by Treiber et al ^28^ for laminitis recurrence in ponies with the pre‐laminitic metabolic syndrome and similar to those reported by Carter et al ^29^ for laminitis recurrence based on basal [insulin]. Similarly, Menzies‐Gow et al ^5^ reported an increased risk associated with increased basal [insulin] and low [adiponectin] in a similar cohort of nonlaminitic ponies.

The current study used a modified OST as a means of assessing the insulin response to oral carbohydrate ingestion. Preliminary data indicated that [insulin] 30 and 60 min after 0.15 ml/kg (low dose) corn syrup administration differed between laminitis prone and never‐laminitic ponies when tested without prior fasting.^30^ In the current study, the dose of corn syrup was increased to 0.3ml/kg to provide greater β‐cell stimulus and thus greater discrimination between hyper‐responsive and normal ponies. Data published subsequent to the current study commencing suggest that 0.45 ml/kg corn syrup may have provided even greater discrimination.^31^ Similarly, a longer sampling period to include responses between 75 and 120 min with more frequent sampling to capture both peak insulin and the area under the insulin curve may have captured additional, potentially important information such as ponies in which peak insulin occurred later, or those in which insulin clearance was delayed.^32^ However, this would have been less practical to apply in the field.

The association between evident divergent hoof growth with subsequent clinically apparent laminitis development is interesting. Divergent hoof growth is common in clinical laminitis cases^20^ and cases of the Equine Metabolic Syndrome/insulin dysregulation^33^ although it is unclear whether it is specific for laminitis (that may be subclinical). The aetiology of divergent hoof growth is unclear, although speculatively insulin‐mediated effects on cell proliferation and differentiation are plausible. The association reported in the current study also supports the hypothesis that divergent hoof growth is a preclinical/subclinical laminitis indicator that may progress to clinically apparent disease.^1^ Divergent hoof growth is an appealing risk marker for clinically apparent laminitis as its assessment is convenient and most ponies’ feet are examined frequently by a farrier. The current study did not investigate how frequently evidence of divergent growth preceded a clinically apparent episode of laminitis nor the risk factors for the development of divergent growth. These warrant further investigation. A weakness of the current study was the subjectivity of the grading scale used to assess divergence and further research is also required to develop repeatable objective grading systems.

Of the statistically significant factors in the initial models, the exercise composite score variable was the only directly modifiable factor. This variable combined the reported exercise frequency and intensity into a single parameter. Whether exercise itself is protective or whether it is simply a marker for other factors associated with lower risk is unclear. Nevertheless, regular low‐intensity exercise with dietary restriction increased insulin sensitivity and had greater benefits than dietary restriction alone in obese equids.^34^ Increased exercise is therefore likely to be a useful strategy for ponies at increased laminitis risk.

Increasing age was a laminitis risk factor in the initial model of owner questionnaire and signalment data. However, it was not an independent factor in the combined models, consistent with age being a marker for other factors, such as insulin dysregulation. Increasing age is associated with greater β‐cell responses.^34^ Similarly, the presence of hypertrichosis was associated with laminitis in the initial models but was not an independent factor in the combined models when a wider set of variables were considered.

The lack of an independent association between hypertrichosis and laminitis in the combined models is consistent with the separate analysis of [ACTH] as a risk factor. The association between [ACTH] and laminitis was weak and only present on univariable analysis of autumn samples. Autumn [ACTH] was a marker for the risk associated with [insulin] (basal or T60). Thus, PPID is unlikely to be an independent risk factor for laminitis but rather a marker of animals that are more likely to have insulin dysregulation (consequent on their age or of PPID). The association between PPID and insulin dysregulation requires further research. The seasonal effect in the current study (in which autumn but not spring [ACTH] showed an insulin‐dependent association with laminitis risk) may relate to increased *pars intermedia* activity in the autumn that increases [ACTH] and the concentrations of ACTH fragments that cross react with the ACTH assay used.^6^ It should also be noted that ponies receiving drug treatment for PPID were not included in the cohort and therefore some more advanced PPID cases will have been excluded possibly biasing the results.

The lack of, or only weak, independent statistical associations between factors such as [ACTH], body condition score or cresty neck score and laminitis does not mean that such factors should be ignored, as the lack of independent association may be due to collinearity with [insulin]. While [insulin] may be a more effective laminitis predictor, if such factors are modifiable and causative of insulin dysregulation rather than simply markers thereof, then they may still be useful intervention targets.

The current study has several limitations. Multivariable clinical prediction models must be interpreted with caution, particularly when there are a large number of potential predictor variables and relatively few events. The existence of a single “true” clinical prediction model is extremely unlikely. The current study prespecified several initial models with very limited numbers of variables selected on the basis of previous data and clinical experience and therefore is at significant risk of bias. Alternative approaches may have yielded better prediction models, however, data‐driven and iterative variable elimination has received significant criticism, in particular in relation to overfitting and optimism.^15^ The current study included limited iterative variable selection in order to strike an appropriate balance. The univariable models included as supplementary material illustrate the associations that may exist between laminitis and a range of factors that could be used for clinical prediction. The study was also limited by the potential for covariates to change between the data collection visits and laminitis occurrence, missing data and data integrity when owner‐provided, the potential for owners or veterinary surgeons to miss mild cases of laminitis or to mis‐diagnose other conditions as laminitis, and for a wide range of other uncontrolled factors to affect the observed data. However, all of these factors are also relevant to clinical practice and the study aim was to identify applicable clinical prediction factors for general practice.

A limitation of the analysis and reporting was that the estimated cumulative incidences are reported over a 4‐year period. This may suggest that a single blood sample predicts laminitis development 4 years later. However, the estimates are based on repeated 6 monthly sampling and it may be more appropriate to interpret the cumulative incidences on the hypothetical basis that the measured parameter would be unchanged over the time at risk. The extent to which features of insulin dysregulation vary within an individual over time^35^ and therefore the frequency with which repeat sampling may be useful requires further research.

Finally, the current study results relate to the specific geographical area studied and the analytical assays used. The ponies studied were kept in groups of at least five animals by experienced owners. The results may not apply entirely to other management methods or sample assays (insulin in particular). Ideally, the results should be validated on another population before being extrapolated more widely.

In conclusion, information available from clinical examination, blood analysis or from owner's accounts of management are all associated with laminitis risk. Risk appears to be concentrated in a minority of ponies that are best identified by basal or oral sugar test stimulated serum insulin concentrations. These values may be used to classify ponies as high‐, medium‐ or low‐laminitis risk such that preventative strategies can be targeted on those at highest risk.

## CONFLICT OF INTERESTS

EJ Knowles is employed by CVS Ltd and provides diagnostic laboratory services through Axiom Veterinary Laboratories. He has received fees for speaking/CPD material from Boehringer Ingelheim. PA Harris is employed by MARS Petcare UK.

## AUTHOR CONTRIBUTIONS

E. Knowles, N. Menzies‐Gow, P. A. Harris and J. Elliott contributed to the study design. E. Knowles was responsible for data collection, study execution and data integrity and had full access to all of the data. E. Knowles and Y. M. Chang analysed the data. All authors contributed to data interpretation, manuscript preparation and approved the final manuscript.

## ETHICAL ANIMAL RESEARCH

The study was approved by the Royal Veterinary College Animal Welfare and Ethical Review Board (2015‐5128) and was conducted under a UK Home Office Licence (PPL 70/8195/ PED 1AA054).

## INFORMED CONSENT

Informed owner consent was obtained for all ponies included in the study.

### PEER REVIEW

The peer review history for this article is available at https://publons.com/publon/10.1111/evj.13572.

## Supporting information

Table S1

Table S2

Table S3

Data S1

Method S1

## Data Availability

The data that support the findings of this study are available from the corresponding author upon reasonable request.
