# Supplementary material for: Predictors of laminitis development in a cohort of nonlaminitic ponies
Source: Equine Vet J. 2022 Apr 1;55(1):12–23. doi: 10.1111/evj.13572 (PMC10084125; doi:10.1111/evj.13572)
Supplement: Supplementary file 1 — Table S1 [file EVJ-55-12-s004.pdf]

Table S1: All variables extracted from the database and considered for further analysis. Variables selected for the initial multivatiable models are highlighted in bold.

Variable name	Description
Signalment, data collection and outcome	
**Age**	Age in complete years at the time of data collection as reported by the owner (birth dates considered as 1 January)
**Breed**	Breed, as reported by the owner was divided into 4 groups:<br>1) Welsh or Welsh Cross<br>2) Shetland, miniature Shetland or Shetland Cross<br>3) Cob or Cob Cross<br>4) All others including unknown
**Sex**	Mare, gelding or stallion, recoded as male or female for analysis
Blood Analytes	
[ACTH]	Basal plasma ACTH concentration (pg/ml)
**ACTH_positive**	ACTH ≥ the middle of the seasonally adjusted equivocal range for PPID diagnosis (≥40pg/ml in spring, ≥75pg/ml in autumn)
**[Adiponectin]**	Basal plasma adiponectin concentration (µg/ml)
**[Glucose]**	Basal plasma glucose concentration (mmol/l)
**[Insulin]T0**	Basal serum insulin concentration (µIU/ml)
[Insulin]T30<br>Rescaled (divided by 10) for some analysis = [insulin]T30/10	Serum insulin concentration (µIU/ml) 30 minutes after dosing oral 'Karo' syrup 0.3ml/kg. (µIU/ml)
[Insulin]T60<br>Rescaled (divided by 10) for some analysis = [insulin]T60/10	Serum insulin concentration (µIU/ml) 60 minutes after dosing oral 'Karo' syrup 0.3ml/kg. (µIU/ml)
Delta Insulin	Absolute 60 minute change in insulin = [Insulin]T60 – [Insulin]T0. (µIU/ml). NB values <0 were set to 0
AUC Insulin<br>Rescaled (divided by 10) for some analysis = AUC Insulin/10	Area under the OST insulin curve =<br>0.25x ([Insulin]T0 + 2x [Insulin] T30 + [Insulin]T60). (µIU/ml.hr)
**[Triglycerides]**	Basal plasma triglyceride concentration (mmol/l)

Morphometric and clinical examination parameters	
bcs_neck	The body condition score for each region 1-9 as described by Henneke et al. [1].
bcs_withers	
bcs_shoulder	
bcs_ribs	
bcs_loin	
bcs_tailhead	
**Body condition score**	Arithmetic mean of BCS scores (1-9) from the neck, withers, shoulder, ribs, loin and tailhead
**Cresty neck score**	0-5 subjective description of the size of the nuchal crest as described by Carter et al. [2].
Weight	Total body mass measured using a portable weighbridge (Equestrian Weigh Platform, Equestrian Products) (kgs).
**Height**	Height measured with a height stick (Shires Extending Measuring Stick, Shires Equestrian) on the first occasion the subject was examined (cms).
Body length	The distance from the most cranial palpable aspect of the point of the shoulder to the most caudal palpable aspect of the ischium (cms).
Neck length	Distance from the most lateral palpable aspect of the transverse process of C1 to the most cranial palpable aspect of the withers (cms).
Neck circumference	The circumference of the neck taken midway along the length (described above) and perpendicular to the dorsal midline of the neck (cms)
Heart girth	The circumference of the thorax running around from just behind the elbow, over the back just caudal to the withers (cms).
Belly girth	The circumference of the abdomen at its widest point (cms).
BMI	Body mass index $=\text{Weight}/(\text{height}/100)^2$
Nc h	Neck circumference to height ratio
Nc nl	Neck circumference to neck length ratio
Hg h	Heart girth to height ratio
Bg h	Belly girth to height ratio
BCI	A body condition index – derived to correspond to body fat percentage. $((\text{heart girth}^{1.18}+ \text{belly girth}^{0.98}+\text{neck circumference}^{1.31})/(\text{height}^{1.23} + \text{body length}^{1.01}))^{5.1}$ Equation obtained by personal communication, Simon Bailey
**Hypertrichosis**	Subjective evidence of hypertrichosis based on veterinary assessment. Subjects that had recently been clipped extensively for winter exercise could not be assessed (present/absent)

Bulging supraorbital fatpads_vet	Subjective evidence of a convex contour to the supraorbital fossa based on veterinary assessment (present/absent)
Pot belly_vet	Subjective evidence of a pot belly, defined as significant ventral deviation of the ventral abdomen caudal to the xiphisternum on veterinary assessment (present/absent)
**Hoof divergence score**	Score for total hoof divergence. Sum of scores for left fore and right fore. 0-4. Evidence of divergent hoof growth was determined by examining the lateral aspect of both front feet (after cleaning/brushing), divergent growth was considered to be present if any of the following conditions were met: circumferential growth rings that were wider at the heel than the toe, horn tubules or pigmented stripes that could be seen to be growing in a curve rather than straight or if the dorsal hoof wall was concave. Each front foot was assessed separately and given a score of 0- no divergent growth, 1 evidence of mild divergent growth only evident on closer examination or 2 marked divergent growth apparent without closer examination. Scores for each foot were added to give an overall divergence score.
Owner Questionnaire	
Yard type	Main use of yard<br>1 = Private yard/owner's home, 2= riding school/riding school + livery yard, 3= charity/rescue centre
Yard size	Number of horses/ponies on the yard:<br>1-5, 6-10, 11-20, 21-50, >50
Main use	Main use of pony:<br>1= general riding, 2= pet/retired, 3= competition, 4= breeding, 5= other<br>Re coded to 1= exercised/ridden or 2= not exercised (pet/retired/breeding)
Turnout time	Hours of daily turnout at time of data collection:<br>0= none, 1= ≤2 hours, 2= 3-6 hours, 3= 7-12 hours, 4= >12 hours, 5= all the time<br>Imputed as 'all the time' if the data were missing but it was confirmed that the pony was always kept at pasture
Turnout cover	Owners' assessment of the extent of grass cover in the turnout area at the time of data collection.<br>0= no grass, 1= very bare, 2= patchy, 3= ok, 4= good/plentiful
Grass length	Owners' assessment of grass length in the turnout area at the time of data collection. 0n= no grass, 1= <5cm, 2= 5-15cm, 3= 15-30cm, 4= >30cm

Grass richness	Owners assessment of the grass richness in the turnout area at the time of data collection: 0= no grass, 1= poor, 2= medium, 3= good, 4= very rich
**Turnout composite score**	Composite score: turnout time + turnout cover (NA if either blank)
Grazing restricted	Owner report of whether grazing is restricted (e.g. grazing muzzle) at the time of data collection
Forage_type	Owner report of the main type of forage (if provided) at the time of data collection: 0= none, 1= dry hay, 2= soaked hay, 3= haylage, 4= straw, 5= silage
Exercise hours	Owner report of the number of hours of weekly exercise at the time of data collection: 0= none, 1= <1h, 2= 1-2h, 3= 2-4h, 4= 4-6h, 5= 6-8h, 6>8h<br>Imputed as zero if the data were missing and the pony was known to be kept as a pet/retired
Exercise_trotting	Owner report of the number of hours trotting per week at the time of data collection: 0= none, 1= <1h, 2= 1-2h, 3= >2h<br>Imputed as zero if the data were missing and the pony was known to be kept as a pet/retired
Exercise_type	Owner report of the intensity of exercise at the time of data collection: 0= no exercise, 1= gentle, 2= medium, 4= fairly hard, 5= hard
**Exercise composite score**	Composite variable: exercise hours + exercise type<br>(NA if either blank)
Expect_to_compete	Owner report of whether the pony is expected to compete in the 3 months following data collection: yes or no.<br>Imputed as no if the data were missing and the pony was known to be kept as a pet/retired
Condition_month	Owners perception of condition change in month prior to data collection: 1= lost weight, 2= stayed about the same, 3= gained weight
Condition_now	Owner’s assessment of condition at time of data collection: 1= very thin, 2= a bit thin, 3= about right, 4= a bit fat, 5= very fat
**Footsore after trimming**	Owners’ perception of a history of being footsore after foot trimming/ shoeing during the 3 months prior to data collection.
Other_recent_illness	Any other illnesses reported by owner over the 6 months prior to data collection
Lethargy	Owner perception of the presence of absence of any of respective clinical sign (all signs of PPID):<br>Lethargy or lack of energy

<table>
<tr><td>Bulging_supraorbital
_fatpads_ow</td><td rowspan="5">Supraorbital fat (fat bulging around the eyes)
long abnormal coat or delayed winter coat shedding
repeated infections
muscle wastage
PUPD- drinking or urinating excessively/</td></tr>
<tr><td>Long_coat</td></tr>
<tr><td>Repeated_infections</td></tr>
<tr><td>Muscle_wastage</td></tr>
<tr><td>Pupd</td></tr>
<tr><td>Ppid_2_or_more</td><td>The presence or absence of two or more signs of PPID reported by the owner</td></tr>
</table>

References

1. Henneke DR, Potter GD, Kreider JL, Yeates BF. Relationship between condition score, physical measurements and body fat percentage in mares. *Equine Vet. J.* 1983;15:371–372.

2. Carter RA, Geor RJ, Burton Staniar W, Cubitt TA, Harris PA. Apparent adiposity assessed by standardised scoring systems and morphometric measurements in horses and ponies. *Vet. J.* 2009;179:204–210.
