# Supplementary material for: Predictors of laminitis development in a cohort of nonlaminitic ponies
Source: Equine Vet J. 2022 Apr 1;55(1):12–23. doi: 10.1111/evj.13572 (PMC10084125; doi:10.1111/evj.13572)
Supplement: Supplementary file 2 — Table S2 [file EVJ-55-12-s002.pdf]

Table S2: Data distribution and complete rate (before imputing any missing data).

Variable	Complete rate (%)	Mean	Standard deviation	25th centile	Median	75th centile
age	96.7%	14.0	6.3	9.0	13.0	18.0
**Blood analytes**						
acth	99.3%	53.3	97.0	19.5	30.5	50.8
acth positive	99.3%	Negative = 1554 Positive =301				
adiponectin	98.1%	17.4	12.0	9.0	14.5	23.3
glucose	98.3%	4.7	0.6	4.4	4.7	5.0
insulinT0	98.8%	23.5	41.9	7.0	13.0	25.0
insulinT30	95.8%	56.3	64.8	21.9	38.7	66.5
insulinT60	95.0%	71.8	91.6	23.2	42.0	82.1
delta_insulin	94.9%	48.5	65.7	12.8	27.5	57.3
aucinsulin	94.5%	52.3	62.7	19.9	34.1	61.45
triglycerides	98.6%	0.3	0.2	0.2	0.3	0.4
**Morphometric features**						
overall_BCS	99.9%	5.7	1.0	5.0	5.7	6.3
Height	99.7%	126.7	17.3	118.0	130.0	140.0
body_length	98.9%	149.2	20.1	138.0	152.0	164.0
neck_length	98.9%	50.5	8.4	45.0	51.0	57.0
neck_circum	98.6%	82.0	11.1	75.0	82.0	90.0
heart_girth	98.9%	159.7	19.8	148.0	162.0	174.0
belly_girth	98.5%	183.3	22.1	170.0	186.0	199.0
Weight	99.6%	331.6	112.2	251.8	336.0	416.0
nc_h	98.3%	0.7	0.1	0.6	0.6	0.7
nc_nl	98.6%	1.6	0.2	1.5	1.6	1.8
hg_h	98.6%	1.3	0.1	1.2	1.3	1.3
bg_h	98.2%	1.5	0.1	1.4	1.4	1.5
Bci	98.1%	13.3	6.0	9.2	11.9	16.0
bmi1	99.3%	199.2	30.1	178.1	199.3	219.7

Body condition score, cresty neck score and hoof divergence score

Variable	Count for each level										
	0	1	2	3	4	5	6	7	8	9	Complete rate (%)
CNS	206	509	596	382	157	3					99.2%
BCS Neck			1	80	250	645	503	285	91	11	99.9%
BCS Withers			6	47	190	559	631	293	124	16	99.9%
BCS Shoulder				26	147	513	633	407	112	28	99.9%
BCS Ribs			2	42	196	628	527	320	126	25	99.9%
BCS Loin			4	22	412	629	468	236	87	8	99.9%
BCS Tailhead			1	27	137	577	617	353	117	35	99.8%
Divergence Score	1085	180	533	9	26						98.1%

Questionnaire variables

Variable	Complete rate	Factor counts
Breed	80.4% (excluding 'unknown')	Welsh or Welsh X: 540<br>Shetland or Shetland X: 279<br>Cob or Cob X:260<br>Other 423 Unknown: 366
Sex	99.1%	Male: 1129<br>Female: 723
yard_number	100%	See table below

yard_type	100%	Private yard: 130<br>Riding school: 1465<br>Charity: 273
yard_size	100%	1-5: 42<br>6-10: 62<br>11-20: 64<br>20-50:1062<br>>50: 638
main_use	98.9%	General riding: 1369<br>Pet/retired: 358<br>Competition: 34<br>Breeding:29<br>Other:58
turnout_time	89.2%	None: 71<br>1-2h: 134<br>3-6h: 167<br>7-12h: 67<br>>12h: 132<br>all the time: 1095
turnout_cover	83.2%	No: grass:505<br>Very bare:185<br>Patchy:218<br>Ok: 437<br>Good/plentiful:210
grass_length	83.1%	No: grass: 505<br><5cm: 687<br>5-15cm: 321<br>15-30cm: 39<br>>30cm: 1
grass_richness	79.4%	No: grass: 505<br>Poor: 278<br>Medium:471<br>Good:217<br>Very rich: 13

turnout_composite	83.2%	0:71<br>1:75<br>2: 63<br>3:50<br>4:114<br>5:333<br>6:147<br>7:186<br>8:346<br>9:21<br>10:149
grazing_restricted	66%	No:1181<br>Yes: 52
forage_type	80.7%	None: 198<br>Dry hay: 924<br>Soaked hay:93<br>Haylage:158<br>Other:135
exercise_hours	85.4%	None:397<br><1h:118<br>1-2h: 93<br>2-4h:262<br>4-6h:234<br>6-8h:252<br>>8h:240
exercise_trotting	80.7%	None:441<br><1h:185<br>1-2h:328<br>>2h:553
exercise_type	85.2%	No exercise: 397<br>Gentle: 231<br>Medium: 650<br>Fairly hard: 313<br>Hard: 1

exercise_composite	84.1%	0: 397<br>1:0<br>2: 93<br>3:61<br>4:93<br>5:172<br>6:239<br>7:231<br>8:186<br>9: 99
expect_to_compete	79.2%	No: 1236<br>Yes: 244
condition_month	82.5%	Lost weight: 237<br>Stayed about the same:1056<br>Gained weight: 248
condition_now	82.6%	Very thin: 4<br>A bit thin: 121<br>About right: 885<br>A bit fat: 452<br>Very fat: 81
footsore_trimming	80.3%	No: 1466<br>Yes: 33
other_recent_illness	81.5%	No: 1409<br>Yes: 113

PPID related variables

PPID related factor or ordinal variables		
Variable	complete rate	Factor counts
Veterinary Assessed		
acth_positive	99.3%	Negative: 1554 Positive: 301
bulging_supraorbital_fatpads_vet	88.3%	No: 1401 Yes: 249
hypertrichosis_vet	87.3%	No: 1583 Yes: 48
pot_belly_vet	87.8%	No: 1379 Yes: 261
Owner Assessed		
Lethargy	79.6%	No: 1473 Yes: 14
bulging_supraorbital_fatpads_ow	79.6%	No: 1481 Yes: 6
long_coat	79.6%	No: 1442 Yes: 45
muscle_wastage	79.6%	No: 1463 Yes: 24
ppid_2_or_more	79.6%	No: 1472 Yes: 15
Pupd	79.6%	No: 1475 Yes: 12
repeated_infections	79.6%	No: 1486 Yes: 1

Numbers of ponies and ponies x sampling times per yard.

yard number	total ponies x sampling times	number of individual ponies
1	42	11
2	26	6
3	91	18
4	127	22
5	56	17
6	22	7
7	119	19
8	108	24
9	117	20
10	74	16
11	81	13
12	90	20
13	88	12
14	117	21
15	16	10
16	15	4
17	66	12
18	29	5
19	51	15
20	169	36
21	124	21
22	47	11
23	82	12
24	111	22
Total	1868	374
