# Supplementary material for: Predictors of laminitis development in a cohort of nonlaminitic ponies
Source: Equine Vet J. 2022 Apr 1;55(1):12–23. doi: 10.1111/evj.13572 (PMC10084125; doi:10.1111/evj.13572)
Supplement: Supplementary file 3 — Table S3 [file EVJ-55-12-s005.pdf]

Equine Veterinary Journal BEVA

Table S3: Univariable associations between laminitis hazard and all variables extracted from the database (ordered by p value (ascending)). CI = confidence interval

Variable	Hazard Ratio (95% CI for HR)	p.value (Wald)
[Insulin]T60$_{10}$	1.1 (1.1-1.1)	2x10^{-23}
AUC insulin$_{10}$	1.1 (1.1-1.1)	3x10^{-20}
[Insulin]T30$_{10}$	1.1 (1.1-1.1)	4x10^{-20}
Deltainsulin	1 (1-1)	3x10^{-17}
[Insulin]T0	1 (1-1)	2x10^{-11}
[Adiponectin]	0.88 (0.84-0.93)	8x10^{-7}
Hoof divergence_score	1.9 (1.4-2.5)	4x10^{-6}
Yard type (Private yard/home = reference)		2x10^{-5}
Riding school/riding school + livery yard	0.2 (0.09-0.46)	0.0001
Charity/rescue centre	0.75 (0.31-1.8)	0.5
Condition_now	2.6 (1.7-4.2)	3x10^{-5}
[Triglycerides]	2.5 (1.6-4.1)	0.0001
Exercise composite score	0.83 (0.74-0.92)	0.0004
Exercise_type	0.58 (0.43-0.79)	0.0005
Exercise_hours	0.76 (0.65-0.89)	0.0006
Exercise_trotting	0.65 (0.5-0.85)	0.002
BCS_loin	1.5 (1.1-1.9)	0.003
[Glucose]	1.7 (1.2-2.5)	0.004
BCI	1.1 (1-1.1)	0.004
Cresty neck score	1.5 (1.1-1.9)	0.007
Main_use not exercised = pet/retired/breeding (reference = exercised)	2.3 (1.3-4.4)	0.007
Nc_h (neck crest: height)	190 (3-12000)	0.01
Bulging_supraorbital_fatpads_vet (vet assessed)	2.4 (1.2-4.8)	0.01
BCS_withers	1.4 (1-1.8)	0.02
Hypertrichosis (vet assessed)	3.3 (1.2-9.5)	0.02
BCS_neck	1.3 (1-1.7)	0.02
Body condition score	1.4 (1-1.9)	0.03
BCS_tailhead	1.3 (1-1.7)	0.04
Yard_size	0.75 (0.55-1)	0.06
Age	1 (1-1.1)	0.08
Hg_h (heart girth: height)	20 (0.69-560)	0.08
Sex	1.7 (0.92-3.1)	0.09
BCS_shoulder	1.3 (0.96-1.6)	0.1
BMI	1 (1-1)	0.1
Grass_richness	1.3 (0.93-1.7)	0.1

Variable	Hazard Ratio (95% CI for HR)	p.value (Wald)
Pot_belly_vet (vet assessed)	0.45 (0.14-1.5)	0.2
Grass_length	1.3 (0.86-2)	0.2
Turnout_cover	1.1 (0.93-1.4)	0.2
Bg_h (Belly girth: height)	4.7 (0.35-62)	0.2
Nc_nl (Neck circumference: neck length)	2.3 (0.53-10)	0.3
Turnout composite score	1.1 (0.95-1.2)	0.3
Neck_circum	1 (0.99-1)	0.3
Height	0.99 (0.98-1)	0.4
ACTH_positive	1.4 (0.56-3.46)	0.5
Muscle_wastage	2.1 (0.28-16)	0.5
Long_coat	1.6 (0.38-6.9)	0.5
Other_recent_illness	1.4 (0.44-4.7)	0.5
Turnout_time	1.1 (0.87-1.3)	0.5
Expect_to_compete	0.76 (0.29-2)	0.6
Condition_month	1.2 (0.65-2.2)	0.6
Breed (Welsh/Welsh X = reference)		0.6
Shetland/Shetland X	0.77 (0.3-2)	0.6
Other/Unknown	0.64 (0.31-1.34)	0.2
Cob/Cob X	1.07 (0.45-2.52)	0.9
BCS_ribs	1.1 (0.82-1.4)	0.6
Grazing_restricted	1.5 (0.19-12)	0.7
Belly_girth	1 (0.98-1)	0.7
Body_length	1 (0.98-1)	0.8
Forage type (reference = no additional forage)		0.8
dry hay	0.53 (0.18-1.54)	0.2
soaked hay	$2x10^{-8}$(0-inf)	>0.9
Haylage	0.52 (0.13-2.07)	0.4
Other	0.35 (0.065- 1.88)	0.2
Weight	1 (1-1)	0.9
Heart_girth	1 (0.98-1)	0.9
Neck_length	1 (0.96-1)	>0.9
ACTH	1 (1-1)	>0.9
Footsore_trimming	$1.1x10^{-7}$ (0-Inf)	>0.9
Lethargy	$3x10^{-7}$ (0-Inf)	>0.9
Bulging_supraorbital_fatpads_ow	$8.2x10^{7}$ (0-Inf)	>0.9
PPID_2_or_more	$3x10^{-7}$ (0-Inf)	>0.9
PUPD	$3x10^{-7}$ (0-Inf)	>0.9
Repeated_inf	$1.7x10^{-5}$ (0-Inf)	>0.9
