# Supplementary material for: Predictors of laminitis development in a cohort of nonlaminitic ponies
Source: Equine Vet J. 2022 Apr 1;55(1):12–23. doi: 10.1111/evj.13572 (PMC10084125; doi:10.1111/evj.13572)
Supplement: Supplementary file 4 — Data S1 [file EVJ-55-12-s001.pdf]

Equine Veterinary Journal BEVA

Supplementary Item 1: Owner/carer questionnaires: the initial questionnaire was used when each pony initially entered the cohort, the follow-up questionnaire at each subsequent visit.

Laminitis Risk in Southern England Initial Questionnaire

Yard and Pony details

1) **Pony name:** ____________________

2) **Owner name:** ____________________

3) **How long have you owned/cared for the pony?** ____________________

4) **Pony age** _______ years estimated/documented (select one)

5) **Breed (if known)**___________________________

6) **Sex:** (select one) Mare/Gelding/Stallion

7) **What is the pony's main current use?** (select one)
General riding/Pet or retired/
Competition (please specify main type and level ________________________________) /Breeding/
Other (please specify) ____

8) **Yard Type?** (select all that apply)
Riding school/Livery yard (please specify Full livery/part livery /DIY) /
Charity rescue centre/ Private yard or Home/ Other (please specify)_______________

9) **How many horses or ponies are kept at the yard?** (select one)
0-5/6-10/11-20/ 21-50/ more than 50

10) **Do you know of any veterinary conditions your pony has suffered from in the past?** (i.e. times the vet has needed to visit or advise other than for routine vaccinations and teeth rasping) please indicate roughly how long ago each condition last occurred

____

____

THIS QUESTIONNAIRE IS PRINTED ON BOTH SIDES OF THE PAPER

Stabling, Turnout and Diet

11) How much turn out does your pony usually have each day at the moment?
(select one)
None/2 hours or less /3-6 hours /7-12 hours /more than 12 hours /Out all the time

12) At the moment if the pony is turned out is this usually: (select one)
During the day/Overnight/Day and night

13) What sort of turn-out does the pony have? (select one)
No grass i.e. dirt paddock, menage or woodchip paddock **(if so skip to question 19)**
Newly sown (in last 5 yrs) grass paddock
Mature grass paddock
Rough grazing (including marshes, moorland and hills)
Other (please specify) _________________________

14) At the moment how well is the turn-out area covered by grass? (select one)
The grass cover is:
Very bare/Patch/OK/Good-plentiful

15) At the moment how long is most of the grass in the turn out area? (select one)
Less than 5cm (2 inches)
5-15cm (2-6 inches)
15-30cm (6-12 inches)
More than 30cm (12 inches)

16) At the moment how rich do you think the grass in the turn out area is?
The grass is: poor/medium/good/very rich

17) When was the field last fertilised? (select one)
At least 10 years ago or never/in the last 5-10 years/in the last 2-5 years/in the last year/don't know

18) Is your pony's grazing restricted in any way e.g. grazing muzzle, strip grazing?
Yes/No (if Yes please specify) ____

19) Has your pony's turnout time changed much or has the field type changed in the last 3 months (e.g. changed from stabled overnight to out 24/7, or movement onto an ungrazed field)**?**
Yes/No/Don't know

20) Do you plan to change your pony's turnout time or field type in the next 6 months?
Yes/No/Don't know

21) Does your pony currently receive any of the following (circle all that apply)
Hay dry/soaked hay/ _____________haylage/straw/silage/Other (please specify)____
If a commercial _________________haylage please specify the type _

22) Does your pony currently receive hard feeds (i.e. commercially manufactured feeds including chaffs/forages)?
Yes once daily/Yes twice daily/No/Other (please specify)_________________

23) If yes please circle all hard feed types that your pony currently receives and if possible list the product names and amounts:
Chaff/Mix/Bran/Unmolassed sugar beet/Molassed sugar beet/pony nuts/
fibre pencils/Oats or Barley/
Other (please specify) ____
Product names and amounts (if possible) ____
____
____
____

24) Please list any other things such as dietary supplements your pony receives: please list the product and approximate amount:
____
____

25) Has the forage (i.e. hay/haylage etc) or the hard feeds in your pony's diet changed in the last 3 months?
Yes/No/Don't know

26) Do you plan to change the forage or the hard feeds in your pony's diet in the next 6 months?
Yes/No/Don't know

PLEASE TURN OVER

Exercise:

27) How many hours a week is your pony exercised at the moment? (select one)
None (retired)/None (temporary rest e.g. injury or out of season)/1 hour or less/1-2h/2-4 hours/4-6 hours/6-8 hours/more than 8 hours

28) Roughly how many hours a week of trotting exercise does your pony have?
None/Less than 1/1-2/more than 2

29) If your pony is exercised what is the main type of exercise? (select one)
Gentle exercise (mostly walk)/medium exercise (mostly walk and trot)/fairly hard work (including trotting, canter/gallop or jumping)/hard fitness work.

30) Do you expect to compete your pony in the next 3 months? Yes/No/Don't know
If yes please specify type and level of competition ____________________________

Condition

31) Over the last month do you think your pony has: (select one)
lost weight/gained weight/stayed about the same?

32) At the moment do you think your pony is: (select one)
very thin/a bit thin/about right/a bit fat/very fat?

33) Over the next month do you expect your pony to: (select one)
Lose weight/gain weight/stay about the same weight

Feet and farriery

34) Before our visit roughly when was the pony last trimmed or shod? (select one)
Less than 1 week ago, 1-3 weeks, 3-6 weeks, 6-9 weeks, more than 9 weeks

35) Does your pony have shoes (nailed or glued on)? (select one)
Yes –in front/Yes- on all feet/No

36) If YES are these conventional metal shoes
Yes/No (if NO please specify what they are) __________________________________

37) Over the last 3 months has your pony been footsore after shoeing/trimming?
(select one) Yes/No/Don't know

38) Over the last 3 months has your pony had any foot problems?
Yes/No/Don't know (if yes please specify: ____________________________________

Health and Medication

39) Has your pony received any medication (other than vaccinations and worming) during the last 6 months?
Yes/No/Don't know (if yes please give brief details):

____

40) Has your pony suffered from any illness or injury (other than laminitis) during the last 6 months?
Yes/No/Don't know (if yes please give brief details):

____

41) Has your pony shown any of the following (tick all that apply)
Lethargy or lack of energy ☐/muscle wastage☐/Supraorbital fat (fat bulging around the eyes)☐/ /drinking or urinating excessively☐/long abnormal coat or delayed winter coat shedding☐/ repeated infections☐

42) Is your pony's worming determined by worm egg counts?
Yes/No/Don't know

43) If no, how often is your pony usually wormed? (select one)
Once a year/twice a year/3-4 times a year, more than 4 times a year

Laminitis in Southern England Follow-Up Questionnaire

1) **Has your pony's use changed or has the pony moved yards in the last 6 months?**
Yes/No (if yes please give details)____
____

2) **During the last 6 months do YOU think your pony has suffered from laminitis?**
Yes/No/Don't know

If NO or DON'T KNOW please skip to the next page

If YES,

3) **Who diagnosed or confirmed the laminitis** (circle all that apply)
I did (pony owner or carer)/a farrier/a vet/
other equine professional (please specify)____________________________

4) **When did the laminitis start?**
____

5) **What do you think the cause was?**
____

6) **Compared with when the laminitis started is the pony?** (please select one)
Sound or back to normal/improved but not back to normal/about the same/worse/put to sleep because of laminitis/Other (please specify)

IF THE LAMINITIS HAS <u>NOT</u> BEEN CONFIRMED BY A VET PLEASE CONTINUE TO ANSWER THE QUESTIONS BELOW. IF IT HAS BEEN CONFIRMED BY A VET THEN YOU DO NOT NEED TO ANSWER ANY FURTHER QUESTIONS.

THIS QUESTIONAIRE IS PRINTED ON BOTH SIDES OF THE PAPER

Stabling, Turnout and Diet

7) How much turn out does your pony usually have each day at the moment?
(select one)
None/2 hours or less/3-6 hours/7-12 hours/more than 12 hour /Out all the time

8) At the moment if the pony is turned out is this usually: (select one)
During the day/Overnight/Day and night

9) What sort of turn-out does the pony have? (select one)
No grass i.e. dirt paddock, menage or woodchip paddock **(if so skip to question 9)**
Newly sown (in last 5 yrs) grass paddock
Mature grass paddock
Rough grazing (including marshes, moorland and hills)
Other (please specify) ___________________________

10) At the moment how well is the turn-out area covered by grass? (select one)
The grass cover is:
Very bare/Patchy/OK/Good-plentiful

11) At the moment how long is most of the grass in the turn out area? (select one)
Less than 5cm (2 inches)
5-15cm (2-6 inches)
15-30cm (6-12 inches)
More than 30cm (12 inches)

12) At the moment how rich do you think the grass in the turn out area is?
The grass is: poor/medium/good/very rich

13) When was the field last fertilised? (select one)
At least 10 years ago or never/in the last 5-10 years/in the last 2-5 years/in the last year/don't know

14) Is your pony's grazing restricted in any way e.g. grazing muzzle, strip grazing?
Yes/No (if Yes please specify) ____

15) Has your pony's turnout time changed much or has the field type changed in the last 3 months (e.g. changed from stabled overnight to out 24/7, or movement onto an ungrazed field)**?**
Yes/No/Don't know

16) Do you plan to change your pony's turnout time or field type in the next 6 months?
Yes/No/Don't know

17) Does your pony currently receive any of the following (circle all that apply)
Dry hay/soaked hay/haylage_______ /straw/silage/Other (please specify)___
If a commercial haylage please specify the type.

18) Does your pony currently receive hard feeds (i.e. commercially manufactured feeds including chaffs/forages)?
Yes once daily/Yes twice daily/No/Other (please specify)_________________

19) If yes please circle all hard feed types that your pony currently receives and if possible list the product names and amounts:
Chaff/Mix/Bran/Unmolassed_____________ sugar beet/Molassed sugar beet/pony nuts/fibre pencils/Oats or Barley/Other (please specify) _______
Product names and amounts (if possible) ____
____
____
____

20) Please list any other things such as dietary supplements your pony receives: please list the product and approximate amount:
____
____

21) Has the forage (i.e. hay/haylage etc) or the hard feeds in your pony's diet changed in the last 3 months?
Yes/No/Don't know

22) Do you plan to change the forage or the hard feeds in your pony's diet in the next 6 months?
Yes/No/Don't know

Exercise:

23) How many hours a week is your pony exercised at the moment? (select one)
None (retired)/None (temporary rest e.g. injury or out of season)/1 hour or less/1-2h/2-4 hours/ 4-6 hours/6-8 hours/more than 8 hours

24) Roughly how many hours a week of trotting exercise does your pony have?
None/Less than 1/1-2/more than 2

25) If your pony is exercised what is the main type of exercise? (select one)
Gentle exercise (mostly walk)/medium exercise (mostly walk and trot)/fairly hard work (including trotting, canter/gallop or jumping), hard fitness work.

26) Do you expect to compete your pony in the next 3 months? Yes/No/Don't know
If yes please specify type and level of competition ___________________________

PLEASE TURN OVER

Condition

27) Over the last month do you think your pony has: (select one)
lost weight / gained weight / stayed about the same?

28) At the moment do you think your pony is: (select one)
very thin / a bit thin / about right / a bit fat/ very fat?

29) Over the next month do you expect your pony to: (select one)
Lose weight / gain weight / stay about the same weight

Feet and farriery

30) Before our visit roughly when was the pony last trimmed or shod? (select one)
Less than 1 week ago, 1-3 weeks, 3-6 weeks, 6-9 weeks, more than 9 weeks

31) Does your pony have shoes (nailed or glued on)? (select one)
Yes –in front/Yes- on all feet/No

32) If YES are these conventional metal shoes
Yes/No (if NO please specify what they are) ________________________________

33) Over the last 3 months has your pony been footsore after shoeing/trimming?
(select one) Yes/No/Don't know

34) Over the last 3 months has your pony had any foot problems?
Yes/No/Don't know (if yes please specify: __________________________________

Health and Medication

35) Has your pony received any medication (other than vaccinations and worming) during the last 6 months?
Yes/No/Don't know (if yes please give brief details):

____

36) Has your pony suffered from any illness or injury (other than laminitis) during the last 6 months?
Yes/No/Don't know (if yes please give brief details):

____

37) Has your pony shown any of the following (tick all that apply)
Lethargy or lack of energy ☐/muscle wastage☐/Supraorbital fat (fat bulging around the eyes)☐/ drinking or urinating excessively☐/long abnormal coat or delayed winter coat shedding☐/ repeated infections☐

38) Is your pony's worming determined by worm egg counts?
Yes/No/Don't know

39) If no, how often is your pony usually wormed? (select one)
Once a year/twice a year/3-4 times a year, more than 4 times a year
