# Supplementary material for: Predictors of laminitis development in a cohort of nonlaminitic ponies
Source: Equine Vet J. 2022 Apr 1;55(1):12–23. doi: 10.1111/evj.13572 (PMC10084125; doi:10.1111/evj.13572)
Supplement: Supplementary file 5 — Method S1 [file EVJ-55-12-s003.pdf]

Methods S1: Sample handling and analysis.

Sample handling

Immediately after collection samples with anticoagulant were chilled in a polystyrene box with ice packs. Clot activator tubes were kept at ambient temperature to allow clotting (or temporarily kept in a pocket if ambient temperatures were cold). On the day of sample collection serum or plasma was separated by centrifugation (10 minutes, 2000g in an unrefrigerated centrifuge), aliquoted and frozen to -20°C for short term storage (usually for one week). Samples were then transferred to a freezer at -80°C for longer term storage prior to analysis.

Sample analysis

[ACTH] was measured by chemiluminescent immunoassay (ACTH Immulite 1000, Siemens) validated for use in the horse [1]. Samples above the linear range (>1250pg/ml) or below the limit of detection of the assay (<10pg/ml) were assigned values of 1250pg/ml and 10pg/ml respectively. [Adiponectin] was measured using a immunoturbidometric assay (ADPN Adiponectin immunoturbidometric assay, catalogue #AO2999, Randox Laboratories) validated for equine samples[2]. [Triglycerides] were measured using a colorimetric method (Prestige 24i TG, P.Z Cormay SA) on an automated spectrophotometer (Biolis 24i premium, Tokyo Boeki) validated in the author's practice laboratory. [Glucose] was measured using an enzymatic colorimetric method performed and previously validated at the Royal Veterinary College diagnostic laboratory. [Insulin] was measured using an automated immunofluorescent assay (IRI Insulin, Tosoh AIA 360, Tosoh Bioscience) validated in the author's practice laboratory. Values above the linear range of the assay (>350µIU/ml) were diluted with the manufacturer's diluent to obtain a quantifiable result. Briefly, to validate the insulin assay, three pools of surplus serum from animals in the cohort were created with high, medium and low concentrations of endogenous equine insulin. Each pool was divided into five aliquots and analysed daily for five days and on one occasion analysed five times on the same day to calculate inter and intra assay variation [3]. To assess dilutional parallelism the high pool was diluted to four in five, three in five, two in five, one in five, one in ten, one in 20 and one in 40. Finally, 109 surplus samples were analysed concurrently with a previously validated radioimmunoassay [4] to indicate correlation. The low, medium

and high pools of serum yielded results of 14.6µIU/ml, 45.5µIU/ml and 288.2µIU/ml. Inter-assay coefficients of variation for the low, medium and high pools were: 4.9%, 7.4% and 6% respectively to give an overall value (± 95% confidence interval (CI)) of 5.9% (±1.6%). Intra-assay coefficients of variation for the low, medium and high pools were 2.9%, 6.1% and 5.4% giving an overall value (±95% CI) of 5% (±1.9%). The mean recovery on dilution of the high pool was 98.6% (±5.9%) of the expected value. Assay comparison revealed excellent correlation with the previously validated assay (r^2=0.97).

References

1. Perkins GA, Lamb S, Erb HN, Schanbacher B, Nydam DV, Divers TJ. Plasma adrenocorticotropin (ACTH) concentrations and clinical response in horses treated for equine Cushing's disease with cyproheptadine or pergolide. Equine Vet. J. 2002;34:679–685.

2. Menzies-Gow NJ, Knowles EJ, Rogers I, Rendle DI. Validity and application of immunoturbidimetric and enzyme-linked immunosorbent assays for the measurement of adiponectin concentration in ponies. Equine Vet. J. 2018;51:33–37.

3. Bland M. How do I calculate a within-subject coefficient of variation. https://www-users.york.ac.uk/~mb55/meas/cv.htm. 2006. Accessed 20 July 2015.

4. Knowles EJ, Harris PA, Elliott J, Menzies-Gow NJ. Use of the oral sugar test in ponies when performed with or without prior fasting. Equine Vet. J. 2017;49:519–524.
